# Stability and reproducibility of 6013 deep inspiration breath-holds in left-sided breast cancer

**DOI:** 10.1186/s13014-020-01572-w

**Published:** 2020-05-24

**Authors:** D. Reitz, F. Walter, S. Schönecker, P. Freislederer, M. Pazos, M. Niyazi, G. Landry, F. Alongi, E. Bölke, C. Matuschek, M. Reiner, C. Belka, S. Corradini

**Affiliations:** 1 Department of Radiation Oncology, University Hospital, LMU Munich, Marchioninistr. 15, 81377 Munich, Germany; 2 Advanced Radiation Oncology Department, IRCCS Sacro Cuore Don Calabria Hospital, Negrar-Verona, Italy; 3 University of Brescia, Brescia, Italy; 4 Department of Radiation Oncology, University Hospital Düsseldorf, Düsseldorf, Germany

**Keywords:** Surface guided radiation therapy, Breast cancer, DIBH, Intra-breath-hold, Inter-fraction, Stability, Reproducibility, Optical surface scanner

## Abstract

**Purpose:**

Patients with left-sided breast cancer frequently receive deep inspiration breath-hold (DIBH) radiotherapy to reduce the risk of cardiac side effects. The aim of the present study was to analyze intra-breath-hold stability and inter-fraction breath-hold reproducibility in clinical practice.

**Material and methods:**

Overall, we analyzed 103 patients receiving left-sided breast cancer radiotherapy using a surface-guided DIBH technique. During each treatment session the vertical motion of the patient was continuously measured by a surface guided radiation therapy (SGRT) system and automated gating control (beam on/off) was performed using an audio-visual patient feedback system. Dose delivery was automatically triggered when the tracking point was within a predefined gating window. Intra-breath-hold stability and inter-fraction reproducibility across all fractions of the entire treatment course were analyzed per patient.

**Results:**

In the present series, 6013 breath-holds during beam-on time were analyzed. The mean amplitude of the gating window from the baseline breathing curve (maximum expiration during free breathing) was 15.8 mm (95%-confidence interval: [8.5–30.6] mm) and had a width of 3.5 mm (95%-CI: [2–4.3] mm). As a measure of intra-breath-hold stability, the median standard deviation of the breath-hold level during DIBH was 0.3 mm (95%-CI: [0.1–0.9] mm). Similarly, the median absolute intra-breath-hold linear amplitude deviation was 0.4 mm (95%-CI: [0.01–2.1] mm). Reproducibility testing showed good inter-fractional reliability, as the maximum difference in the breathing amplitudes in all patients and all fractions were 1.3 mm on average (95%-CI: [0.5–2.6] mm).

**Conclusion:**

The clinical integration of an optical surface scanner enables a stable and reliable DIBH treatment delivery during SGRT for left-sided breast cancer in clinical routine.

## Introduction

Deep Inspiration Breath-hold (DIBH) is a well-established radiation technique to achieve a significant cardiac dose reduction during adjuvant radiotherapy (RT) in left-sided breast cancer [1–4]. Relative dose reductions of the mean heart dose (MHD) or the left anterior descending (LAD) artery by more than 50% as compared to free breathing (FB) have been reported [4–6]. It has also been shown that even the mean left lung dose is reduced by about 20% on average [7]. As a result, heart-sparing techniques provide a significant risk reduction of major coronary events [8], as the risk increases linearly with the mean heart dose, by about 7.4% per Gy [9]. To date, several different breath-hold techniques are available and used in routine clinical practice. These techniques differ significantly in terms of the utilized devices, intrafraction monitoring and patient feedback systems. In general, a distinction is made between voluntary or computer-controlled DIBH techniques, such as surface-guided or spirometry-based systems, respectively [10–13].

The purpose of the present study was to assess intra-breath-hold stability and breath-hold reproducibility using an automated surface-based technique. In general, intrafraction patient movements during breast cancer radiotherapy could have an impact on adequate dose delivery [14–17]. This fact is even more important during DIBH irradiation, because the target could significantly move out of the radiation fields if the breath-hold is not kept at a stable level. Surface guided radiation therapy (SGRT) offers the possibility to monitor patient movements in real-time using a non-invasive approach, without any additional radiation exposure. SGRT has also proven to be a reliable tool for patient positioning by complementing in-room laser-assisted positioning, as well as for intrafraction monitoring during breast radiotherapy [18–21]. Moreover, SGRT may even reduce the frequency of conventional imaging during breast DIBH RT [22].

The aim of the present study was to analyze intra-breath-hold stability and inter-fraction reproducibility during surface-guided breast radiotherapy in a large study cohort treated with the optical surface imaging system Catalyst™ (C-Rad, Uppsala, Sweden).

## Material and methods

Between February 2017 and October 2018, patients with left-sided breast cancer treated by surface-guided DIBH RT at the Department of Radiation Oncology, University Hospital, LMU Munich were recruited for the present prospective study. RT related information and patient characteristics were retrieved from medical records. Patients were treated either with a conventional fractionated (2Gy in 25 fractions) or a hypofractionated RT scheme (2.67Gy in 15 fractions). If indicated, a sequential boost to the tumour bed was applied with a dose of 5x 2Gy or 8x 2Gy. All treatment plans consisted of 3D conformal RT (3DCRT) plans using two opposing tangential beams for the breast/chest wall, with the addition of 1 to 4 subfields to increase dose homogeneity, as well as anterior/posterior fields for regional nodal irradiation (RNI). The study was approved by the local ethics committee of the University Hospital, LMU Munich (No. 352–16 ex 09/2016) and registered at German Clinical Trials Register (DRKS-ID: DRKS00011407). Written informed consent was obtained from all participants.

### Optical surface scanning system

The Catalyst™ optical surface scanner uses visible light to scan the body surface using three charged-coupled device (CCD) cameras placed in 120-degree angles. Measurements are based on optical triangulation and a three-dimensional surface image of the scanned area is calculated [23]. The system allows monitoring the patient surface by comparing the real-time image with an initially acquired reference image and calculating absolute position deviations from the surface-projected isocenter, using a registration algorithm. The system has a motion detection accuracy of < 0.5 mm for a rigid body. This was verified by physicists of our institution using phantom measurements prior to clinical implementation of the method. A more detailed description of the clinical setting of the present study was reported earlier [4].

### Clinical workflow

**Fig. 1:**
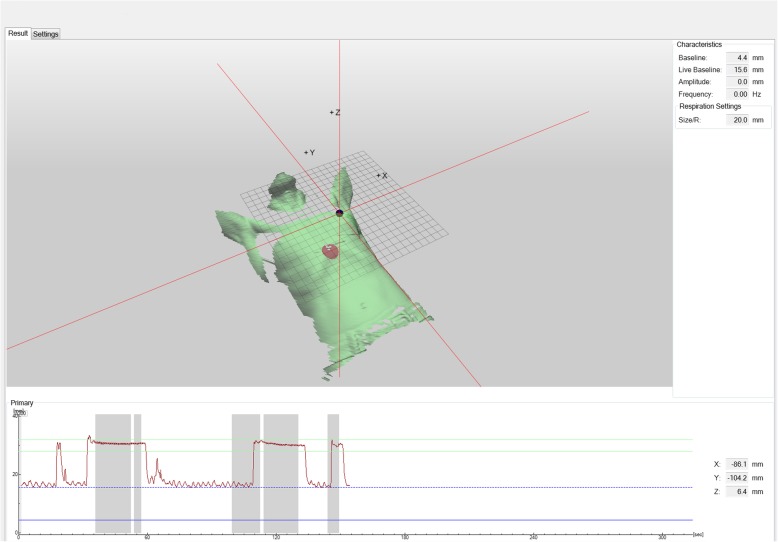
Graphical demonstration of the c4D-Tool; the red point on the patients surface is the reference point for tracking the vertical amplitude during breath-hold; the lower part of the screenshot shows an amplitude over time-plot

**Fig. 2:**
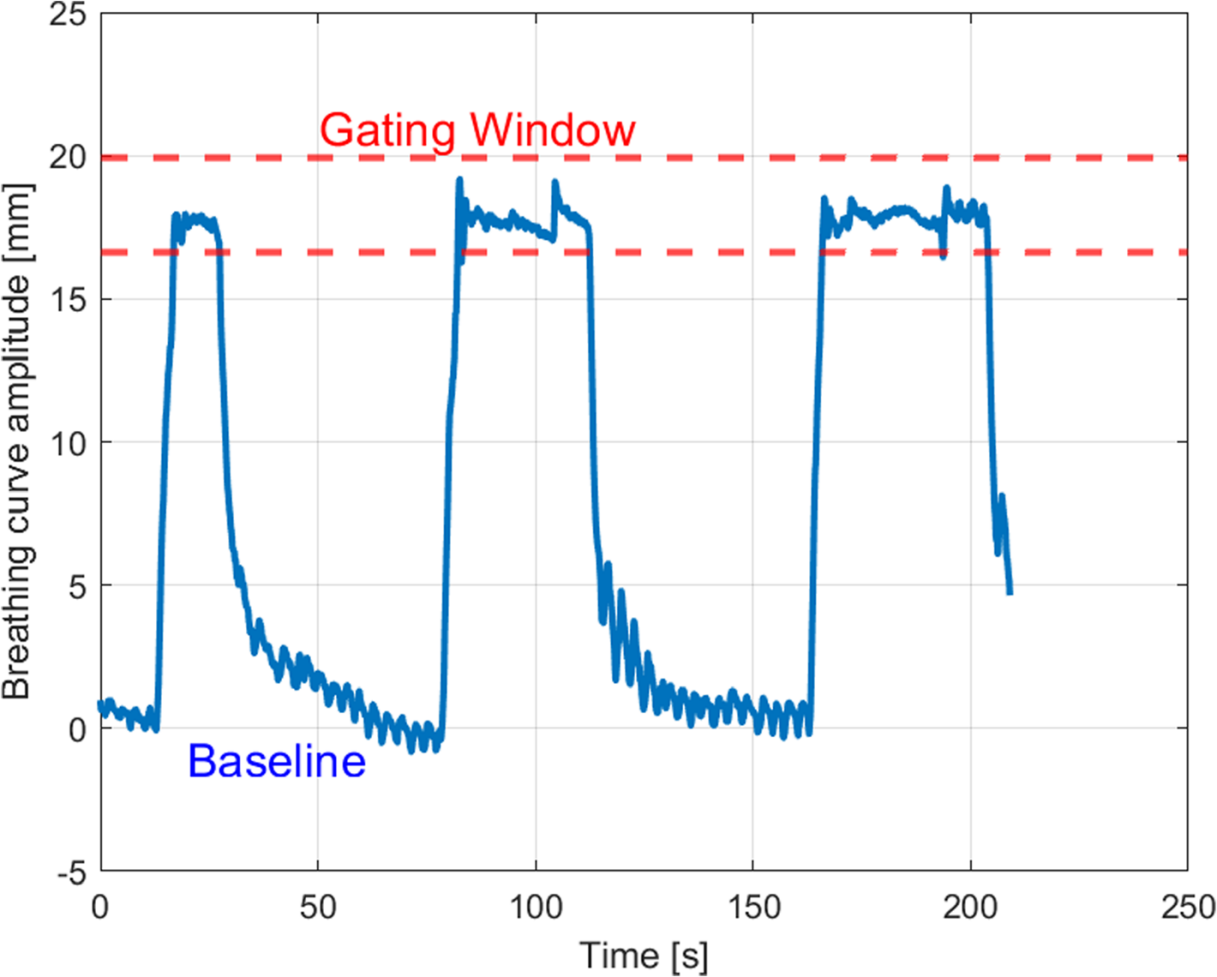
Time plot showing the vertical breathing curve amplitude over time; dashed red lines are showing the lower and upper limit of the gating window

Prior to CT acquisition, the individual breathing baseline (the maximum expiration during FB) of each patient was assessed using the laser-based surface scanner Sentinel™ (C-Rad, Uppsala, Sweden) (see Fig. 1). The gating point was placed on the patient’s xiphoid process. Patients were then asked to perform a comfortable DIBH, without any visual feedback. After a few training repetitions, the gating window was set individually with a maximum height of 4 mm around the patient-specific vertical displacement measured with the Sentinel™, which was defined by the average displacement from all training cycles. The gating window is defined as the area of the respiratory amplitude, where the automated respiratory gating is performed during treatment delivery (beam on, see Fig. 2). The width of the gating window was initially set to 2 mm below and above the breath-hold level and was adjusted individually for each patient. Thereafter, the procedure was repeated using full audio-visual feedback (video goggles) and the simulation CT was performed using a Toshiba Aquillion LB CT Scanner (Toshiba Medical Systems Corporation, Japan) in DIBH. In general, the gating window was determined during the initial simulation CT and was not changed during the course of the treatment. At the linac, patient set-up and DIBH gating was performed using the optical surface scanner Catalyst™. Due to inter-fractional setup variations, a new baseline was always established at the beginning of each treatment fraction and the gating window was automatically set at a relative vertical distance from this baseline by the Catalyst™ software. Several breath-holds were required during DIBH treatment: one breath-hold during each image-guided patient set-up procedure (e.g. portal imaging) and multiple breath-holds for dose delivery.

### Data processing

Surface-based data including real-time measurements of the amplitudes along the vertical direction, the breathing baseline, the width of the gating window and the beam on/off status were retrieved from the Catalyst™ software (Fig. 1). All data were extracted from the Catalyst™ software and analyzed using MATLAB Release 2018b (The MathWorks, Inc., Natick, Massachusetts, United States).

### DIBH stability and reproducibility

**Fig. 3:**
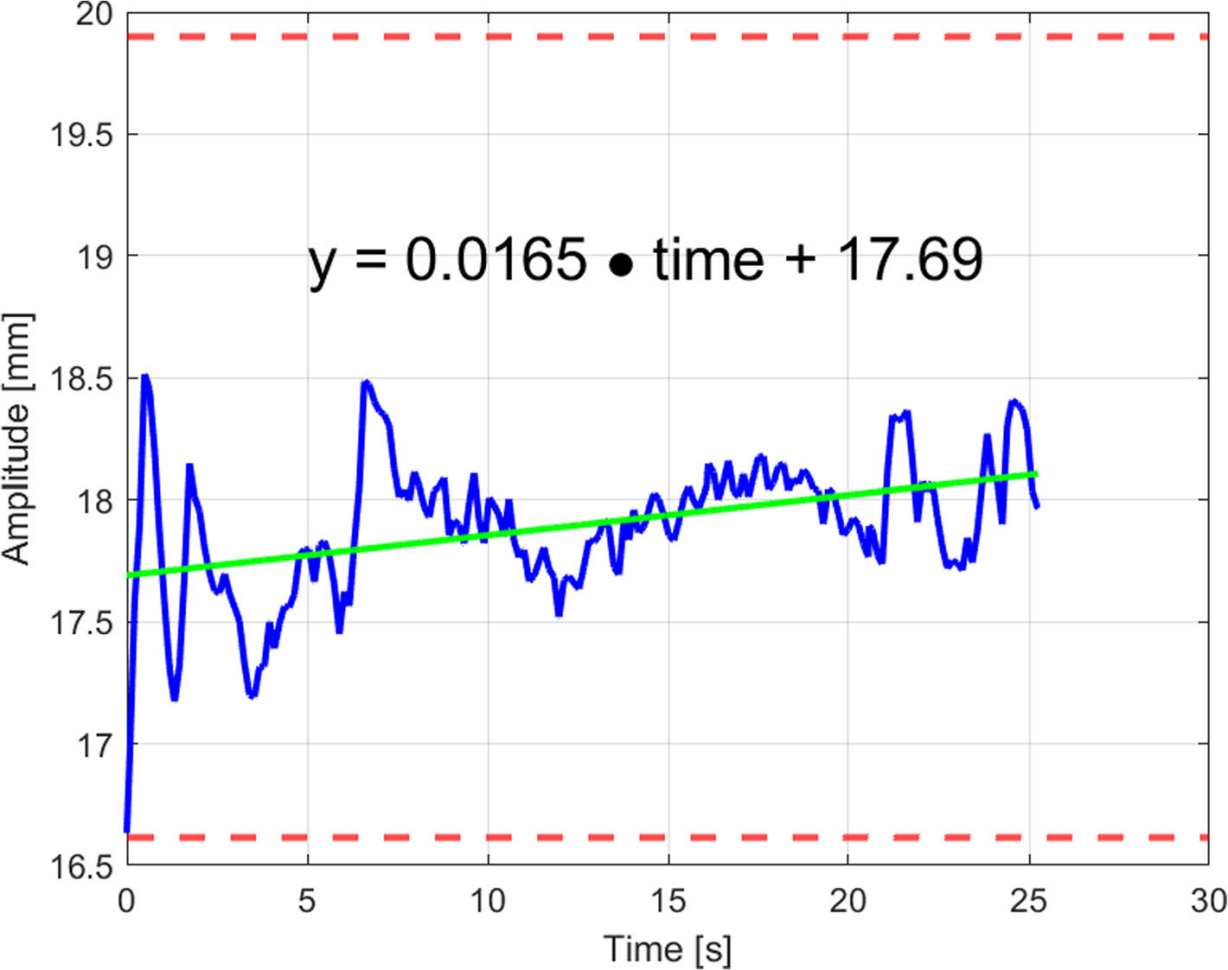
One deep inspiration breath-hold cycle plot showing the vertical deviation in millimeters over time (blue); the green line is a linear fit plot of the blue signal (example of the linear function by linear fit: *y = 0.0165•time+ 17.69*); dashed red lines are showing the lower and upper limit of the gating window

DIBH stability was defined as the amplitude deviation between baseline and the patien-specific gating window (during each breath-hold). Therefore, two parameters were calculated as a measure of stability. The first evaluated parameter was the standard deviation (SD) of the breath-hold level inside the gating window. The second parameter was the linear amplitude deviation during a DIBH maneuver. In this setting, the gradient of a straight line from a linear regression model is multiplied with the DIBH-time interval and results in an amplitude deviation during the DIBH maneuver, as described by Cervino et al. [24]. An example is given in Fig. 3. Since the gradient of the linear amplitude deviation can be either positive or negative, we also used an absolute linear deviation parameter especially for correlation analysis.

Moreover, we analyzed the patient-specific inter-fraction DIBH reproducibility during the entire treatment period (comparing all breath-holds of all treatment fractions). In this context, reproducibility between breath-holds was defined as the consistency between the breathing amplitudes. For this purpose the breathing amplitudes of all breath-holds of a patient were calculated and the difference between the minimum and the maximum value was calculated and used as a measure of reproducibility.

### Statistical analysis

Correlation analysis between different parameters was performed using Spearman’s rank correlation coefficient. Friedman’s test for coupled samples and a Nemenyi-post-hoc-analysis was applied for inter-fraction analysis of DIBH reproducibility. The patient-specific mean DIBH amplitude of all breath-holds during the entire treatment course was analyzed. Since there is no normal distribution for the measured parameters, additional information including median values and 95%-confidence intervals were provided to reliably describe the data distributions. For all statistical analyses a significance level of α = 0.05 was defined. MATLAB was utilized for data extraction, as well as data processing and R 3.3.2 with libraries (R.matlab, ggplot2, plot3D) for statistical analyses.

## Results

### Patient and tumour characteristics

The present study cohort included 103 patients. Most patients had early stage breast cancer, classified pT1 (61/103, 59.2%) or pT2 (25/103, 24.3%) with mainly negative nodal status pN0 (75/103, 72.8%). Overall, 42 patients (40.8%) received a conventional fractionated RT regimen (2Gy in 25 fractions) to the breast or chest wall, 14% received an additional irradiation of the regional lymph nodes and 35% received a sequential boost to the tumour bed (2Gy in 5 or 8 fractions). Table 1 gives an overview including absolute and relative incidences for different clinicopathological and radiotherapy parameters.

**Table 1:** Descriptive patient characteristics and radiotherapy parameters of the study cohort (*n* = 103)

	**No. (%)**
**Age at diagnosis (yrs.)**	
mean ± SD	57.7 ± 11 years
median	58.0 years
range	32–80 years
**Tumour stage**	
pTis	11 (10.7%)
pT1	61 (59.2%)
pT2	25 (24.3%)
pT3	4 (3.9%)
pT4	2 (1.9%)
**Nodal status**	
pN0	75 (72.8%)
pN1	15 (14.6%)
pN2	1 (1%)
pN3	1 (1%)
pNx	11 (10.7%)
**Fractionation**	
Normo-fractionated (2/50 Gy)	42 (40.8%)
Hypo-fractionated (2.67/40 Gy)	61 (59.3%)
**Boost**	
no	67 (65%)
yes	36 (35%)
**Radiotherapy**	
Whole-Breast	98 (95.1%)
Chest-wall	5 (4.9%)

### Descriptive analysis

In summary we analyzed 1944 treatment fractions and 6013 breath-holds during beam-on-time, which corresponds to an average of 3.1 breath-holds during a single treatment fraction. Breath-holds during image guidance (portal imaging, CBCT) were excluded from this analysis. Figure 2 shows a plot of a typical treatment session showing the baseline, the gating window and multiple breath-holds over the duration of a treatment fraction.

**Fig. 4:**
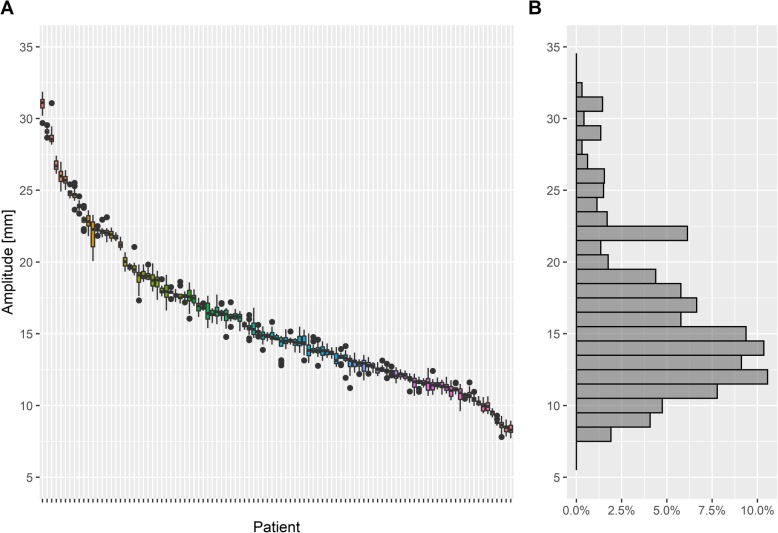
**a** Box plots of breathing amplitudes during breath-hold for every patient including all breath-holds sorted by the median value, **b** histogram of the breathing amplitudes (*n* = 103 patients, 6013 breath-holds)

An overview of the breathing amplitude, defined as the vertical deviation between breathing baseline and mean breath-hold level of one deep inspiration breath-hold within the gating window, is given in Fig. 4a as box plots for all patients (*n* = 103) and all breath-holds (*n* = 6013), sorted in descending order of the patients’ median breathing amplitude. Figure 4b represents the frequency of the breathing amplitudes in a histogram. The breathing amplitude of all patients was 15.8 mm with a SD of ±5.4 mm (95%-CI: [8.5–30.6] mm), the median was 14.7 mm. The mean width of the gating window was 3.5 mm (95%-CI: [2–4.3] mm). In the present cohort, the minimum width was 2 mm, only two patients had a gating window of 5 or 6 mm, as it was more difficult for them to keep a stable DIBH. The mean beam on time during a single breath-hold was 18.4 s (SD 10.1 s; 95%-CI: [6.6–34.4] seconds).

### Breath-hold stability analysis

**Fig. 5:**
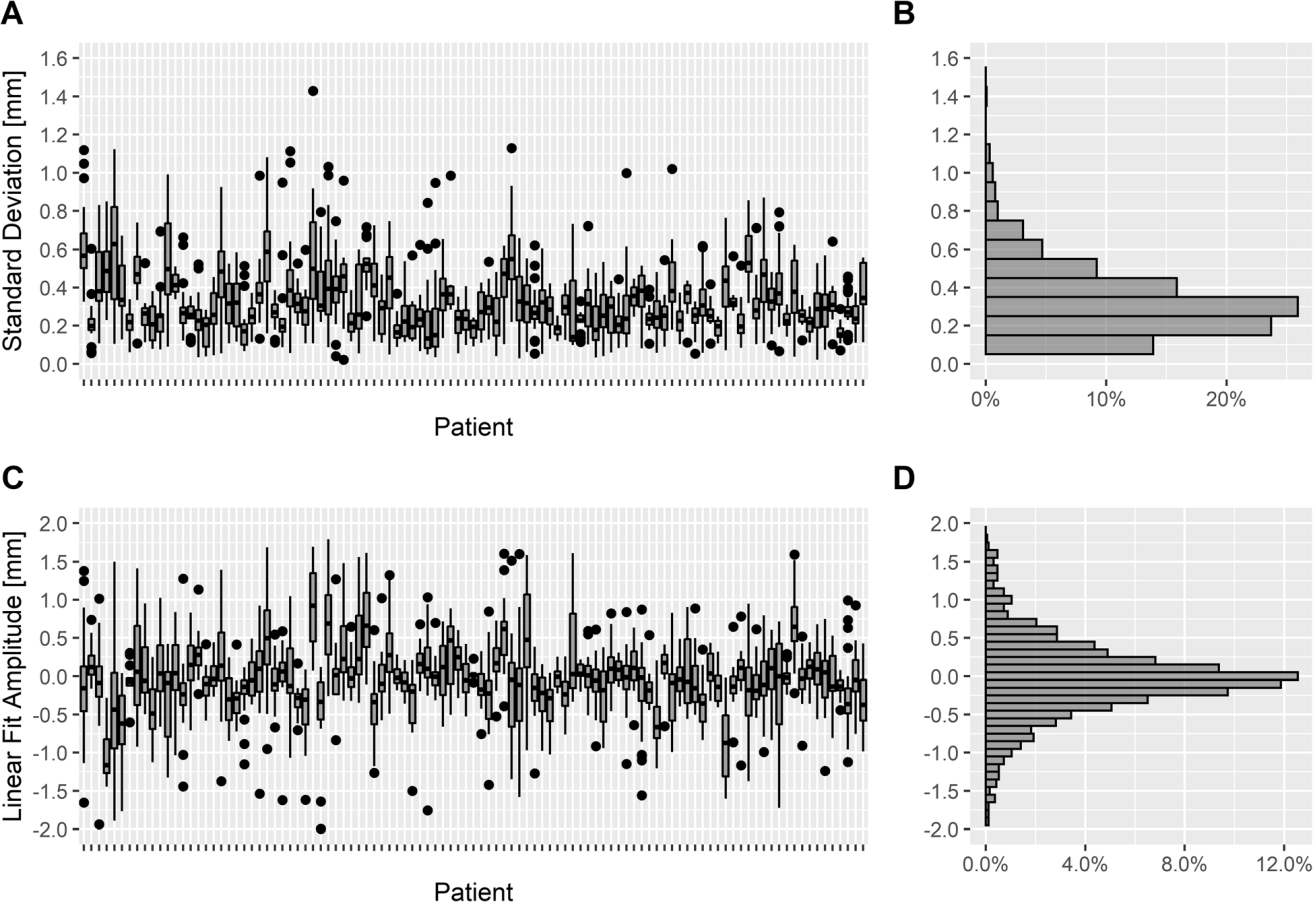
**a** Box plots of standard deviation of the breath-hold levels for every patient. **b** histogram of the standard deviations. **c** Box plots of the linear fit amplitude in millimeters inside the gating window during breath-hold for every patient based on linear fit model. **d** histogram of the linear fit amplitudes (*n* = 103 patients, 6013 breath-holds)

The SD of the breath-hold level during the DIBH showed a value of < 2 mm during all evaluated breath-holds and the median SD of the breath-hold level was 0.3 mm (95%-CI: [0.1–0.9] mm). Most patients had a SD of the breath-hold level of < 1 mm during all of their breath-holds. 95% of all data samples were inside the interval [− 2; + 2] mm around the mean value. Figure 5a shows the SD of the breath-hold level inside the gating window as box plots for each patient and all breath-holds, the frequency of the SD is indicated in a histogram in Fig. 5b. Furthermore, the breath-hold stability was evaluated using linear deviation analysis (Fig. 5c), the frequency of the linear deviation is shown in a histogram in Fig. 5d. The median absolute linear deviation was 0.4 mm (95%-CI: [0.01–2.1] mm), and the mean value was 0 mm (95%-CI: [− 1.6–1.7] mm), indicating that the drift over time inside the gating window was limited.

In order to investigate whether there is a correlation between the median deep inspiration amplitude (gating window) and the median SD of the breath-hold level, we applied Spearman’s rank correlation coefficient between them. The analysis was not statistically significant (r_S_ = 0.1; *p* = 0.4), meaning that the deep inspiration amplitude (gating window) had no effect on the DIBH stability during gating. Furthermore, we compared the SD of the breath-hold level to the linear deviation based on the linear fit model (compare Fig. 5a, c) and observed a statistical significance (r_S_ = 0.6; *p* < 0.001).

### DIBH reproducibility

The difference between the maximum and minimum breathing amplitude across all DIBHs of a patient was calculated, the results were averaged over all patients and showed a mean value of 1.3 mm (95%-CI: [0.5–2.6] mm), the SD was 0.6 mm and the median 1.2 mm. Friedman’s test for coupled samples of the breathing amplitudes of all breath-holds of a patient during the entire treatment course was significant (*p* < 0.001), which means that the breathing amplitudes between individual DIBH maneuvers of a patient during the treatment course were significantly different. In Nemenyi-post-hoc-analyses there were also significant differences between the breathing amplitudes of various DIBH cycles of a patient during all treatment sessions (*p* < 0.001). Nevertheless, the maximum differences of the patient-related breathing amplitudes of all breath-holds during the whole treatment session and averaged over all patients were quite small (mean 1.3 mm). Spearman’s rank correlation was applied between the median deep inspiration amplitude and the maximum difference in the breathing amplitude across all DIBHs of a patient analyzed over all patients (r_S_ = 0.2; *p* = 0.03), additionally we analyzed the correlation between the SD of the breath-hold level and the maximum difference in the breathing amplitude across all DIBHs of a patient over all patients (r_S_ = 0.4; *p* < 0.001).

## Discussion

The present study addresses the topic of DIBH stability and reproducibility during surface-guided left-sided breast radiotherapy. We report on intra-breath-hold stability and inter-fraction reproducibility during DIBH radiotherapy in a large breast cancer patient study population (103 patients and 6013 breath-holds) treated with an automated gating technique using an optical surface scanner. It is known from previous studies, that the Catalyst™ system offers a high level of stability and accuracy in monitoring breast cancer patients during DIBH compared to other tracking and guidance systems [25].

In the present series, the breathing amplitude was 15.8 mm. Regarding DIBH stability, the median SD of the breath-hold level over the entire treatment course was 0.3 mm (95%-CI: [0.1–0.9] mm), highlighting a high degree of stability. This could also be verified by a second stability parameter based on the linear fit model (mean value 0 mm (95%-CI: [− 1.6–1.7] mm)). The SD of the breath-hold level and the stability parameter from the linear fit model showed a significant correlation and can therefore be seen as comparable parameters for the degree of stability (r_S_ = 0.6; *p* < 0.001). There was no correlation between the median deep inspiration amplitude (gating window) and the median SD of the breath-hold level during breath-hold (r_S_ = 0.1; *p* = 0.4), indicating that the DIBH amplitude had no effect on the stability within the gating window. The breathing amplitude between baseline and gating window has limited impact on the analyses of the DIBH reproducibility, as the gating window was individually selected according to the patient’s ability to maintain a stable breath-hold level. Since the beam is only triggered inside the gating window, the breath-hold level inside this window was the main focus of this study.

For inter-fraction reproducibility, the breathing amplitudes between different DIBH-maneuvers of a patient during all treatment fractions were compared and showed significant differences (*p* < 0.001). The absolute differences were quite small (mean 1.3 mm). Our results have shown a significant moderately correlation between the stability parameter SD and the maximum difference in the breathing amplitude across all DIBHs of a patient analyzed over all patients (r_S_ = 0.4; *p* < 0.001), which means that a higher stability leads to a higher reproducibility and vice versa.

Similarly to the present analysis, Xiao et al. analyzed over 7200 DIBH cycles using the surface imaging system AlignRT (VisionRT, London, UK). The same authors found a DIBH-stability of < 0.7 mm and a reproducibility of < 2.2 mm, which is similar to the present results. They analyzed the median of the 5th–95th percentile range of the translational displacement during a single breath-hold or during all breath-holds throughout a single treatment session [26]. Unfortunately, only the vertical amplitude could be evaluated with the Catalyst™ system, which is a limitation of the present study. In contrast, Fassi et al. observed three-dimensional deviations and used spirometry-based monitoring of patients with left-breast DIBH radiotherapy. They analyzed intra-breath-hold, intrafraction and inter-fraction motion with an infrared optical tracking system to assess the variability of the external surface position in all three directions. Taken together, intra- breath-hold stability had a median value of < 1.9 mm in any direction [27].

Similar to this, Kügele et al. have analyzed the intrafraction isocenter reproducibility during DIBH-radiotherapy resulting in a median value of 1 mm in all three possible directions, however, also values up to 5 mm were observed. In the present study regarding DIBH reproducibility, only the difference between the maximum and the minimum breathing amplitude of a patient was calculated, which was around 1.1 mm in median for all patients over the entire treatment course and is similar to the median value of Kügele et al. [28]

Cervino et al. have analyzed the reproducibility and stability of DIBH with and without visual feedback. Reproducibility was defined as the maximum difference between the mean values of individual absolute breathing amplitudes during breath-hold of a patient during one treatment session. The higher the difference between the individual DIBH maneuvers, the worse the reproducibility. The authors concluded that there is a significant difference between the use and omission of video feedback. With video feedback (analogous to our setting) the reproducibility was 0.5 mm and the stability was 0.7 mm [24]. In the present study, the slope inside the gating window and subsequently the drift over time was quite small (median < 0.4 mm), which shows similar results. Stock et al. have shown that the addition of video feedback in DIBH improved the relative reproducibility in over 524 DIBH maneuvers [29]. Therefore, video feedback is a standard procedure at our institution during DIBH radiotherapy.

In a previous published study [19], a large study cohort of breast cancer patients was observed by intrafraction surface monitoring during free breathing RT (without DIBH). All data samples of the vertical amplitude deviation during beam-on-time were averaged over all treatment sessions and all patients, the mean and standard deviation were 0.4 ± 1 mm (95%-CI: [− 1.6–2.6] mm). In the current setting, all patients were treated with DIBH and the median SD of the breath-hold level was much smaller: 0.3 mm (95%-CI: [0.1–0.9] mm). Therefore, the SD of the breath-hold level during DIBH RT is over half the size smaller than the one in free breathing. One relevant factor is that patients were performing breathing maneuvers and were therefore actively paying attention to their breathing patterns. Another important aspect is the video feedback for the patient, which gives the patient an immediate feedback about the breathing excursions so that the patient can react before a relevant deviation occurs [19].

A main advantage of the surface scanning system used in the present study is the automated gating mechanism. If the breath-hold level falls out of the gating window, the irradiation is stopped automatically without the need for any manual intervention. Based on a typical ±2 x SD-interval ([− 2; + 2] mm) that covers over 95% of all values around the mean value, a gating window width of 4 mm seems to be an appropriate choice. Nevertheless, one of the limitations of the present study is that based on a technical limitations only vertical deviations are monitored by the Catalyst™ system, as longitudinal, lateral and rotational deviations are not recorded during gated RT.

## Conclusion

The SGRT system enabled a stable and reliable DIBH treatment delivery in left-sided breast cancer in a large cohort (103 patients, 1944 treatment fractions, 6013 breath-holds). As a measure of DIBH stability, the median SD and absolute linear deviation of the breath-hold level inside the gating window were both < 0.5 mm. Regarding reproducibility of DIBHs, the differences of the breathing amplitudes in all patients and all treatment fractions were 1.3 mm on average.

## Data Availability

Not applicable.

## Abbreviations

CCD: Charged-coupled device
CI: Confidence interval
DIBH: Deep inspiration breath-hold
IMRT: Intensity modulated radiation therapy
MRI: Magnetic resonance imaging
RT: Radiotherapy
SD: Standard deviation
VMAT: Volumetric Arc Therapy
FB: Free breathing
CT: Computed tomography
SGRT: Surface guided radiotherapy
LAD: Left artery descending
MHD: Mean heart dose
CBCT: Conebeam CT

## Glossary

baseline: maximum vertical expiration level during free breathing within one fraction
breath-hold level: vertical amplitude of the breathing curve from baseline during deep inspiration breath-hold
breathing amplitude: vertical deviation between breathing baseline and mean breath-hold level of one deep inspiration breath-hold

## References

[1] Corradini S, Alongi F, Andratschke N (2019). MR-guidance in clinical reality: current treatment challenges and future perspectives. Radiat Oncol.

[2] Pazos M, Schönecker S, Reitz D (2018). Recent developments in radiation oncology: an overview of individualised treatment strategies in breast Cancer. Breast Care (Basel).

[3] Hayden AJ, Rains M, Tiver K (2012). Deep inspiration breath-hold technique reduces heart dose from radiotherapy for left-sided breast cancer. J Med Imaging Radiat Oncol.

[4] Schönecker S, Walter F, Freislederer P (2016). Treatment planning and evaluation of gated radiotherapy in left-sided breast cancer patients using the catalyst(TM)/sentinel(TM) system for deep inspiration breath-hold (DIBH). Radiat Oncol.

[5] Bergom C, Currey A, Desai N (2018). Deep inspiration breath-hold: techniques and advantages for cardiac sparing during breast Cancer irradiation. Front Oncol.

[6] Yu P-C, Wu C-J, Tsai Y-L (2018). Dosimetric analysis of tangent-based volumetric modulated arc therapy with deep inspiration breath-hold technique for left breast cancer patients. Radiat Oncol.

[7] Oechsner M, Düsberg M, Borm KJ (2019). Deep inspiration breath-hold for left-sided breast irradiation: analysis of dose-mass histograms and the impact of lung expansion. Radiat Oncol.

[8] Sripathi LK, Ahlawat P, Simson DK (2017). Cardiac dose reduction with deep-inspiratory breath-hold technique of radiotherapy for left-sided breast Cancer. J Medic Phys.

[9] Darby SC, Ewertz M, McGale P (2013). Risk of ischemic heart disease in women after radiotherapy for breast cancer. N Engl J Med.

[10] Wong JW, Sharpe MB, Jaffray DA (1999). The use of active breathing control (ABC) to reduce margin for breathing motion. Int J Radiat Oncol Biol Phys.

[11] Kubo HD, Len PM, Minohara S (2000). Breathing-synchronized radiotherapy program at the University of California Davis Cancer Center. Med Phys.

[12] Latty D, Stuart KE, Wang W (2015). Review of deep inspiration breath-hold techniques for the treatment of breast cancer. J Med Radiat Sci.

[13] Jensen CA, Abramova T, Frengen J (2017). Monitoring deep inspiration breath-hold for left-sided localized breast cancer radiotherapy with an in-house developed laser distance meter system. J Appl Clin Med Phys.

[14] Pazos M, Fiorentino A, Gaasch A (2019). Dosisvariabilität verschiedener Lymphknotenstationen während der lokoregionalen Bestrahlung bei Mammakarzinom: Einfluss des Luftanhaltens in tiefer Inspiration. Strahlenther Onkol.

[15] Pazos M, Walter F, Reitz D (2019). Einfluss der Patientenpositionierung mittels optischem Oberflächenscanner auf die Verwendung von Verifikationsaufnahmen und die Dauer der Neueinstellung bei Brustkrebspatientinnen. Strahlenther Onkol.

[16] Yue NJ, Li X, Beriwal S (2007). The intrafraction motion induced dosimetric impacts in breast 3D radiation treatment: a 4DCT based study. Med Phys.

[17] George R, Keall PJ, Kini VR (2003). Quantifying the effect of intrafraction motion during breast IMRT planning and dose delivery. Med Phys.

[18] Carl G, Reitz D, Schonecker S (2018). Optical surface scanning for patient positioning in radiation therapy: a prospective analysis of 1902 fractions. Technol Cancer Res Treatment.

[19] Reitz D, Carl G, Schonecker S (2018). Real-time intra-fraction motion management in breast cancer radiotherapy: analysis of 2028 treatment sessions. Radiat Oncol.

[20] Crop F, Pasquier D, Baczkiewic A (2016). Surface imaging, laser positioning or volumetric imaging for breast cancer with nodal involvement treated by helical TomoTherapy. J Appl Clin Med Phys.

[21] Hamming VC, Visser C, Batin E (2019). Evaluation of a 3D surface imaging system for deep inspiration breath-hold patient positioning and intra-fraction monitoring. Radiat Oncol.

[22] Laaksomaa M, Sarudis S, Rossi M (2019). AlignRT® and catalyst™ in whole-breast radiotherapy with DIBH: is IGRT still needed?. J Appl Clin Med Phys.

[23] Freislederer P, Reiner M, Hoischen W (2015). Characteristics of gated treatment using an optical surface imaging and gating system on an Elekta linac. Radiat Oncol.

[24] Cerviño LI, Gupta S, Rose MA (2009). Using surface imaging and visual coaching to improve the reproducibility and stability of deep-inspiration breath-hold for left-breast-cancer radiotherapy. Phys Med Biol.

[25] Kalet AM, Cao N, Smith WP (2019). Accuracy and stability of deep inspiration breath-hold in gated breast radiotherapy - a comparison of two tracking and guidance systems. Phys Med.

[26] Xiao A, Crosby J, Malin M (2018). Single-institution report of setup margins of voluntary deep-inspiration breath-hold (DIBH) whole breast radiotherapy implemented with real-time surface imaging. J Appl Clin Med Phys.

[27] Fassi A, Ivaldi GB, Meaglia I (2014). Reproducibility of the external surface position in left-breast DIBH radiotherapy with spirometer-based monitoring. J Appl Clin Med Phys.

[28] Kugele M, Edvardsson A, Berg L (2018). Dosimetric effects of intrafractional isocenter variation during deep inspiration breath-hold for breast cancer patients using surface-guided radiotherapy. J Appl Clin Med Phys.

[29] Stock M, Kontrisova K, Dieckmann K (2006). Development and application of a real-time monitoring and feedback system for deep inspiration breath-hold based on external marker tracking. Med Phys.

